# How different health literacy dimensions influences health and well‐being among men and women: The mediating role of health behaviours

**DOI:** 10.1111/hex.13208

**Published:** 2021-02-04

**Authors:** Fan Zhang, Peggy P. L. Or, Joanne W. Y. Chung

**Affiliations:** ^1^ Department of Health and Physical Education The Education University of Hong Kong Hong Kong Hong Kong; ^2^ Department of Public Health and Preventive Medicine, School of Medicine Jinan University Guangzhou China; ^3^ School of Nursing and Health Studies The Open University of Hong Kong Hong Kong China

**Keywords:** health behaviour, health literacy dimensions, older adults, physical condition, well‐being

## Abstract

**Background:**

Health literacy, the ability to access, understand, evaluate and apply health information, was found to contribute to positive health outcomes, possibly via promoting healthy behaviours. However, the specific pathways linking different health literacy skills to health and well‐being have remained unclear.

**Methods:**

A cross‐sectional survey with structural questionnaires was administered among 2236 adults in Hong Kong (mean age = 46.10 ± 19.05). Health literacy was measured by HLS‐Asian‐47. Participants' physical conditions and subjective well‐being were predicted by health literacy and health behaviours with structural modelling path analysis.

**Results:**

Health literacy in finding and understanding information showed a direct effect on enhancing physical health, while applying information capacity had an indirect positive effect via promoting health behaviours, which was moderated by sex. Only among women, this indirect effect predicting fewer physical symptoms and better well‐being was significant.

**Conclusions:**

Different health literacy dimensions showed distinct direct and indirect pathways in influencing health for men and women. Based on the findings, skill trainings should be developed to enhance both gender's abilities of finding and understanding health information, while the ability of applying health information should also be improved for modifying lifestyle and promoting health, particularly for women.

**Patient or Public Contribution:**

Two thousand and two hundred thirty‐six adults from different districts of Hong Kong participated in the study, and responded to questions on health literacy, behaviours and health status.

## 1 BACKGROUND

Health literacy, referring to a set of abilities to access, comprehend, appraise and apply information to effectively promote and maintain health in different contexts,^1^ was found to play a key role in individual's health behaviours and health status.^2^, ^3^ However, the prevalence of inadequate health literacy was considerably high, particularly among older adults. For example, the Agency for Healthcare Research and Quality (AHRQ) has found that approximately one third of Americans only has limited health literacy, and this rate went up to 70% among those aged 75 and above.^4^ A recent systematic review on the prevalence of limited health literacy in Southeast Asia has reported that with a large variation across five countries (ie, Laos, Malaysia, Myanmar, Singapore and Thailand), on average, over 50% of the population showed limited health literacy,^5^ and the rate was even higher in healthcare settings (67.5%). Similar results were reported in Hong Kong population by a recent study,^6^ while the prevalence of limited health literacy was even higher in mainland China. In a sample of 1360 participants (aged 15‐69) in Shanghai, the prevalence of limited health literacy was approximately 85%. In consistent, when looking at certain type of health literacy , over 70% of people showed limited health literacy about chronic disease ^7^; and about 80% did not have adequate health literacy about infectious disease.^8^ With such a high prevalence of limited health literacy in the Chinese society, it is possible that the general public has remained unaware about the impact of health literacy.

Previous literature, mostly with western samples, has showed that greater health literacy was consistently associated with various benefits for individual's health, including more healthcare actions,^2^ better health status ^3^ and greater subjective well‐being.^9^ A systematic review reported that limited health literacy was associated with poorer physical health and higher all‐cause mortality rate even after controlling for cognitive functioning.^10^ Inadequate health literacy could also lead to lower medical adherence among patients with cardiovascular disease,^11^ poorer glycaemic control in type‐2 diabetes ^12^ and higher hospital admission.^13^

In addition, health literacy was also found to affect individual's mental health,^14^, ^15^ although the existing evidence has been relatively thin. In the review by Berkman et al, ^10^ only one study showed low health literacy was related to more depressive symptoms after controlling for the confounders.^14^ By investigating the relationship between health literacy and happiness, Angner et al, ^9^ found inadequate health literacy, in addition to poverty and poor health, was associated with lower level of happiness. However, despite this finding being widely cited, the single‐item measurement for health literacy (ie, ‘how confident are you in filling out medical forms by yourself’) may not accurately capture individual's ability to process health‐related information, and the question about happiness is not sufficient to indicate one's well‐being. Therefore, the current study would address the effects of health literacy on both subjective well‐being and physical health.

Another objective of the current study is to explore the underlying mechanism of health literacy. It was suggested that health literacy may benefit health via promoting people's healthy lifestyle and behaviours. Unhealthy behaviours, such as smoking, alcohol consumption or being physically inactive, could contribute to poorer health and are directly linked to the top five causes of death (ie, heart disease, cancer, cerebrovascular disease, respiratory disease and diabetes ^16^). Fortunately, these risk behaviours were modifiable by certain psychosocial factors such as health literacy. With higher level of health literacy, people are more likely to engage in healthy lifestyles, including frequent physical exercise,^17^ reduced usage of alcohol, as well as regular physical examination, etc.^18^, ^19^ However, regarding the relationship between health literacy and dietary habits ^20^ or smoking,^21^, ^22^ the finding bas been rather inconclusive. For example, Geboers et al,^22^ have analysed the data of 3,241 older adults from the LifeLines Cohort Study and found inadequate health literacy was associated with poorer health habits such as limited physical activities, insufficient intake of fruit and vegetables and low alcohol use, but not smoking. This mixed finding might be related to the measures of health literacy. In fact, among the various measurements adopted by the abovementioned studies, few of them have captured the multifaceted nature of health literacy in the analysis. For example, Suka et al, (2015) has measured health literacy as a multi‐dimensional concept (ie, functional literacy, critical literacy and communicative literacy), yet only total score was used in the analysis.^17^ In the studies of Gebeors (2014, 2016), a three‐item questionnaire was adopted to evaluate people's perceived capacity in understanding hospital or medical instructions.^20^, ^22^ However, how the impact of health literacy may vary across different domains of health literacy capacities were rarely covered. As an exception, by addressing the capacities of accessing to, comprehending, applying and evaluating health‐related information, Panahi et al,(2017) found the first three were more important for smoking cessation among college students.^23^ Therefore, instead of general health literacy, the current study examines the relationships between different dimensions of health literacy and health outcomes.

In addition, we also aim to address the potential sex difference in the effects of health literacy. Past research has found women's life expectancy is usually 4‐5 years longer than men, although they are less healthy than men at any age.^24^ When including the sex differences in health behaviours (eg, consumptions of tobacco, alcohol or drug) into the economic model of health deficit accumulation, Schünemann et al,^25^ found an additional 89% of the gender gap in life expectancy was explained. However, where this sex differences in health behaviour arises from has remained unclear, and psychosocial factors such as health literacy may play a major role.^2^, ^26^ Existing literature showed that the level of health literacy was usually lower among men than women (for a review, see ^27^), despite their tendency to over‐report when answering health literacy questions.^28^ Probably because women watch more health‐related television programmes and have greater social engagement, resulting in higher health literacy and healthier lifestyle.^29^ However, no study has examined the link between sex difference in health literacy and health behaviour in predicting health status.

With a sample of 2236 community‐dwelling individuals, the current study has a threefold research purpose: (a) to explore how the effects of different health literacy skills would be mediated by health behaviours; (b) to test whether the mediation pathways of health behaviour differ between predicting physical conditions and subjective well‐being; (c) to clarify whether the indirect effects of health literacy are moderated by sex. Smoking, drinking and physical exercise were selected to indicate people's health behaviour, which are common behaviours in Hong Kong society. Also, these behaviours could reflect gender‐specific preferences, that is the rate of habituated smokers and alcohol users are higher among older male than female, and men tend to have more frequent physical exercise than women.^30^, ^31^, ^32^ In addition to providing an updated profile of health literacy in Hong Kong, the current study also aimed to obtain valuable insights for tailoring educational programmes to promote public health.

## 2 METHODS

### 2.1 Design

A cross‐sectional study was conducted with structured questionnaires among individuals from different districts of Hong Kong. For younger and mid‐age adults, the questionnaires were self‐administered. For those aged 65 or above, the questionnaires were administered by a trained research assistant, in case they may have difficulties in understanding the questions due to the relatively low level of education. Written consent was obtained at the beginning of the study.

### 2.2 Sample

A random sampling was used to recruit two thousand six hundred and thirteen adults from different districts of Hong Kong, with a multi‐age stratified clustered sampling method (ie, including similar number of participants aged 18‐29, 30‐64 and 65 or above). The number of participants from different gender was also balanced. Participants who met the following inclusion criteria were recruited via invitation letters and emails, including the following: (a) aged 18 or above; (b) native Chinese speaker (including both Cantonese and mandarin, with the majority of the participants being Cantonese speakers); (c) no history of cognitive impairment (according to the record provided by the community centre which helped with the recruitment). From the original sample of 2613 participants, 85.6% have completed the survey. The ethical approval for the study was obtained from the Human Research Ethics Committee of Education University of Hong Kong.

### 2.3 Measures

Participants’ demographic information, including sex (0 = male,1 = female), age, education (0= ‘Primary school or lower’, 1= ‘secondary school or above’), marital status (0 = single/divorced/widowed; 1 = married), were collected. Participants also reported their health literacy, health behaviour, physical symptoms and subjective well‐being.

Health literacy (HL) was measured by the Chinese version of HLS‐EU (HLS‐Asian‐Q.^29^ HLS‐Asian‐Q includes 47 items assessing the information‐processing abilities across three domains of health, that is health care, disease prevention and health promotion. Four types of information‐processing abilities were evaluated: (a) the ability to seek and obtain health information (13 items, eg, ‘How difficult it is for you to find out where to get professional help when you are ill’); (b) the ability to understand or comprehend health information (11 items, eg, ‘How difficult it is for you to understand what your doctor says to you’); (c) the ability to appraise, interpret or filter health information (12 items, eg, ‘How difficult it is for you to judge if the information on health risks in the media is reliable’); and (d) the ability to communicate or apply the information to maintain and improve health (11 items, eg, ‘How difficult it is for you to decide if you should take a vaccination’). Participants were asked to choose from ‘1’ (very difficult) to ‘4’ (very easy) when responding to the items, and the response of ‘5’ (do not know) was coded as missing. The average score of subscale was generated to indicate the level of HL in different dimensions.^33^ For general HL, the mean score of all the items was transformed into an HL index ranging from 0 to 50 according to the formula suggested by the European Health Literacy Project [I = (Mean −1) * 50/3],^34^ with an higher score indicating greater HL. Participants were grouped into four levels based on the cutoff value suggested by prior study^35^: an HL index of 0 to 25 indicates the health literacy is ‘inadequate’, and 26 to 33 indicates ‘problematic’; an index from 34 to 42 indicates ‘sufficient’, and above 42 indicates ‘excellent’ HL. HLS‐Asian‐Q has been validated in 6 Asian countries/regions including Taiwan, Indonesia, Kazakhstan, Malaysia, Myanmar and Vietnam.^29^ The Cronbach alpha in our sample was 0.98, suggesting good internal consistency.

Health behaviour was measured by three items asking about individual's smoking, drinking and physical exercise. The responses were recoded into dichotomous variables to indicate whether the participant is a current user of tobacco (0 = ‘non‐smoker’, 1 = ‘smoker’), alcohol (0 = ‘non‐user of alcohol’, 1 = ‘alcohol user’) or a frequent exerciser (0 = ‘frequent exerciser, ie, doing exercise for 30 minutes over 2 times per week’; 1 = ‘infrequent exerciser’). Three items were combined to generate a total score to be used in the pathway analysis, with higher score indicating more presences of unhealthy behaviours.

Individual's subjective well‐being was measured by the 5‐item World Health Organization well‐being index (WHO well‐being index,.^36^ It asked participants in the past two weeks, how often they have ‘felt cheerful and in good spirits’, ‘felt calm and relaxed’, ‘felt active and vigorous’, ‘woken up feeling fresh and rested’ and ‘felt daily life has been filled with interesting things’. A 6‐point response set was used (‘0’ = ‘at no time’, and ‘5’ = ‘all of the time’). The total score of all five items was generated and a higher score indicating better well‐being. Good reliability was indicated by a Cronbach alpha of 0.89.

The presences of twenty‐eight physical symptoms and chronic diseases were asked to evaluate participants’ self‐reported physical health.^37^ It included hypertension, high blood cholesterol, high blood lipid, diabetes, cardiovascular disease, heart failure, respiratory disease, asthma, thyroid disease, liver disease, rheumatism, arthritis, osteoporosis, other musculoskeletal disease, cancer, depression, anxiety disorder, mood disorder, other mental health problem, eating disorder, alcoholism, drug abuse, reproductive disease, hearing impairment, visual impairment, limb loss and other. The total number of ‘yes’ responses was obtained to indicate health condition.

### 2.4 Statistical analyses

**Figure 1:**
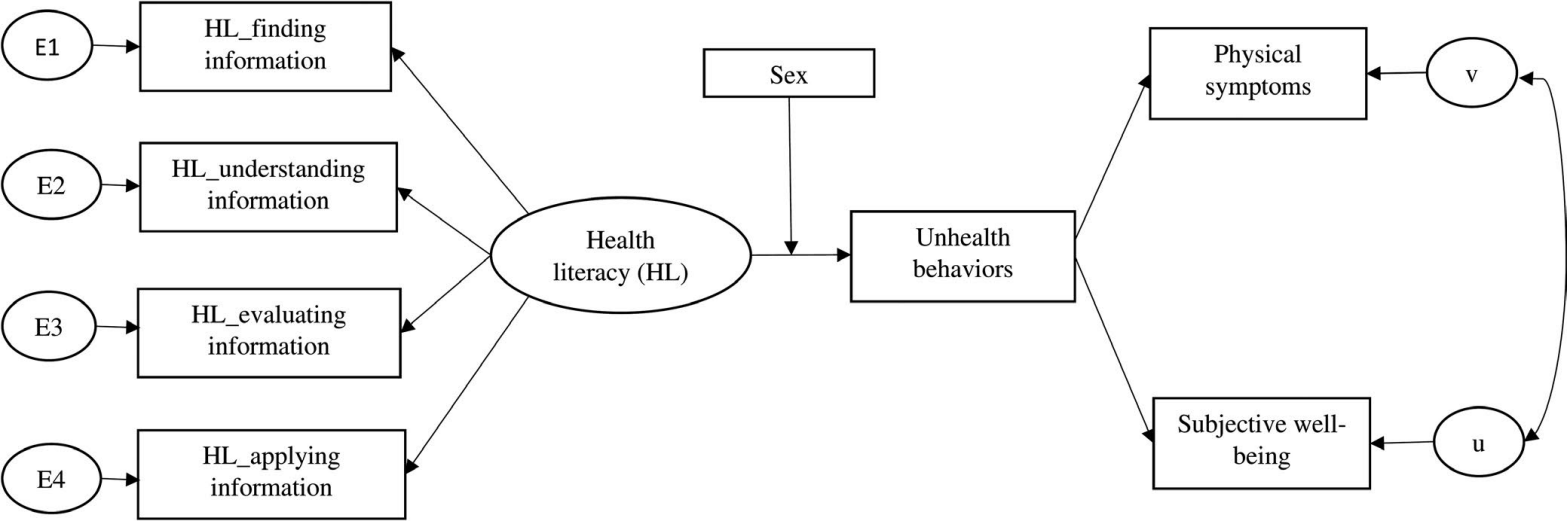
The hypothesized model of health literacy, health behaviours and health outcomes

The Lavaan package in R was used to conduct the structural equation modelling,^38^ to test the hypotheses regarding how different dimensions of health literacy influence physical health and subjective well‐being, as well as whether health behaviour would mediate the associations if there is any. The reason to use structural equation modelling (SEM) is because that the correlation between the two outcomes, physical symptoms and subjective well‐being, should be considered when including them in the pathway model. Age, marital status and education level were controlled as covariates. Pairwise deletion was used to deal with the missing values in the dataset, which only omitted specific variables with missing data on an analysis by analysis basis, to maximize the available data. The scores of four HL capacities were entered as predictors, with the presence of physical symptoms and subjective well‐being index as outcome variables. Since we are interested in investigating how health literacy may differ between men and women, and whether this could further lead to sex difference in health behaviours, sex was included as a moderator in the pathway between health literacy to health behaviour. The hypothesized model was displayed in Figure 1.

## 3 RESULTS

Two thousand and two hundred thirty‐six adults (aged from 18 to 93, mean = 45.07 ± 19.05) participated in the survey. 53.8% of the participants were female and 55.1% were married, with the majority having secondary education or above (79.7%, for details, see Table 1). The average number of reported physical symptoms was 1.20 (SD = 1.56), with no sex difference (*P* = .143). The total score of well‐being was 14.84 (SD = 4.85), and women showed higher level of well‐being than men (*P* = .005). The average number of unhealthy behaviours is 0.91 (SD = 0.90) with men showing more unhealthy behaviours than women (*P* < .001). Robust maximum likelihood was used to deal with the skewed data. By adopting the formula to transform the HL rating, the average HL score was 31.24 (SD = 8.61). Based on this score, participants were categorized into four groups of HL, 20.7% were in the inadequate HL group, 35.2% in problematic HL group, 35% in sufficient HL group and 9.6% reported an excellent level of HL (see Table 1). Since sex was proposed as a moderator, we have tested the sex difference in having limited HL (inadequate and problematic) and adequate HL (sufficient and excellent), and the results showed that there were more women being in the limited HL group compared with men (χ^2^ = 4.23, *P* = .04). Across four domains of HL, evaluating information was perceived as the most difficult (mean per item was 2.76, SD = 0.59), while applying information was perceived as the easiest (mean per item was 2.95, SD = 0.52). No significant sex difference was spotted in different HL domains, although women showed a tendency to perceive evaluating health‐related information more difficult than men (*P* = .059).

**Table 1:** The descriptive results of demographic information, health literacy, health behaviours and health status

	Mean (SD)	Range	No. of Missing
Age	46.10 (19.5)	18‐93	0
Sex (% of women)	53.8%		1
Education (% of having secondary education or above)	79.7%		0
Marital status (% of married)	55.1%		0
Total number of unhealthy behaviours	0.91 (0.9)	0‐3	1
Women	0.72 (0.80)		
Men	1.14 (0.94)		
% of smokers	23.0%		5
% of alcohol users	33.7%		21
% of non‐frequent exercisers	48.3%		7
No. of health conditions	1.13 (1.56)	0‐15	2
Women	1.18 (1.68)	0‐11	
Men	1.18 (1.41)	0‐15	
Well‐being	14.84 (4.85)	0‐25	3
Women	15.10 (4.91)	0‐25	
Men	14.53 (4.75)	0‐25	
Health literacy	31.25 (8.61)	0‐50	7
Insufficient HL (%)	20.7%		
Problematic HL (%)	35.2%		
Sufficient HL (%)	34.5%		
Excellent HL (%)	9.6%		
	Mean per item (SD)		
Finding information	2.88 (0.57)		11
Understanding information	2.92 (0.55)		8
Evaluating information	2.76 (0.59)		14
Applying information	2.95 (0.52)		8

Abbreviations:

HL, health literacy; SD, standardized deviation.

**Figure 2:**
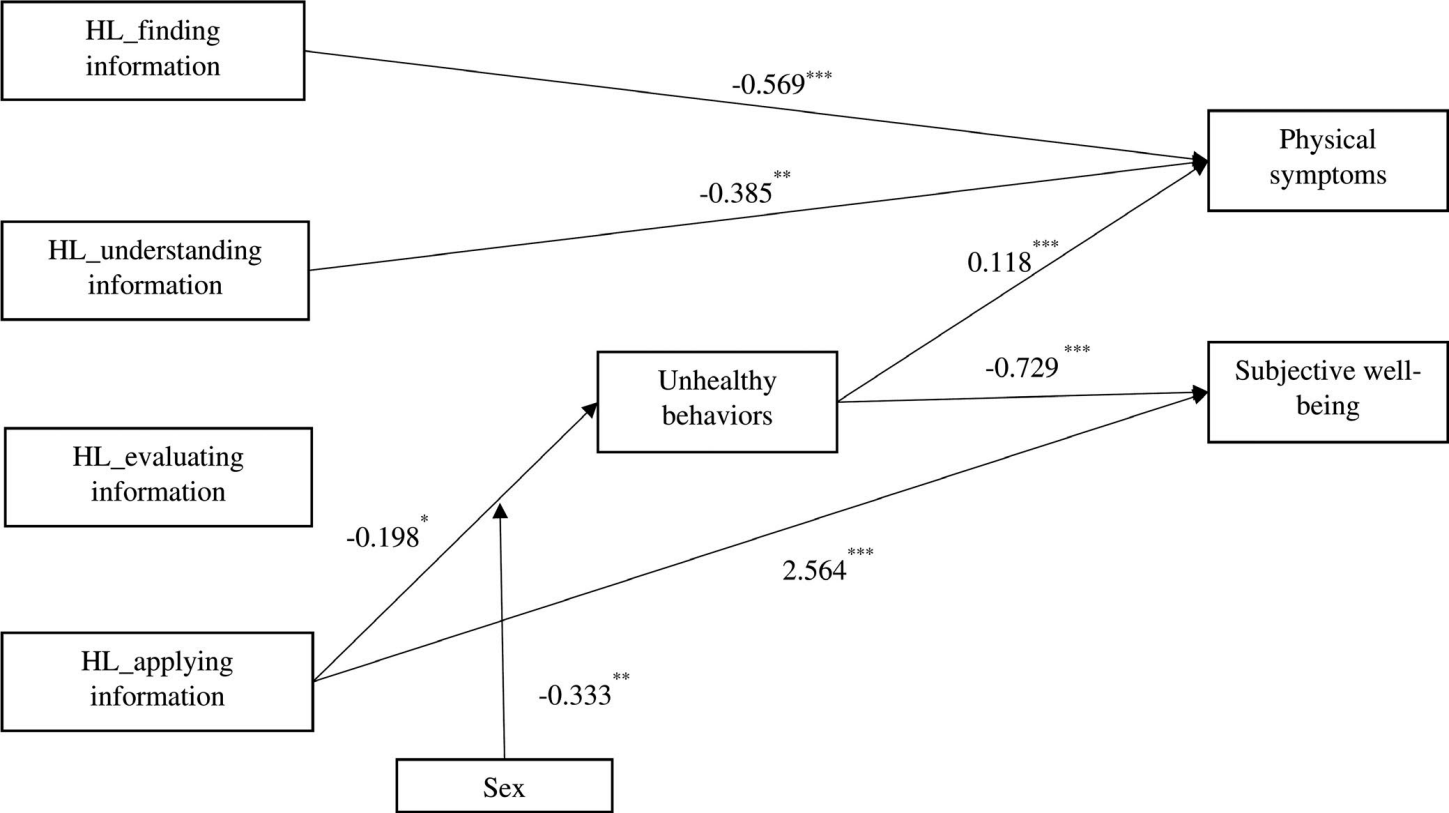
The structural model of health literacy predicting physical health and well‐being (Note: health behaviour, physical symptom and well‐being were adjusted for individual's age, educational level and marital status)

The correlations among predictors, mediator, moderator and health outcomes were displayed in Table 2. Path analysis using structural equation modelling approach was performed to examine the goodness‐of‐fit of the hypothesized path model predicting individual's health outcomes, including physical symptoms and sum score of well‐being. The two outcome variables were regressed on individual's age, education, marital status, sex and HL capacities (ie, finding information, understanding information, evaluating information and applying information). By using the Lavaan package in R, the goodness‐of‐fit of the hypothesized model was considered good (chi‐square value χ^2^ = 28.163, degree‐of‐freedom *df* = 9, *P* = .001, RMSEA = 0.03, SRMR = 0.005, CFI = 0.988, NNFI = 0.952). The structural relationships with standardized path coefficients among the variables are presented in Figure 2 (only significant paths were included).

**Table 2:** The correlation matrix among health literacy, health behaviour and health outcomes

	Education	Marital status	Gender	HL total	HL_finding info	HL_ understanding Info	HL_evaluating info	HL_applying info	Health behaviour	Physical symptom	Well‐being
Age	−0.614**	0.390**	0.049*	−0.304**	−0.348**	−0.315**	−0.248**	−0.213**	−0.146**	0.553**	0.080**
Education	1										
Marital Status	−0.111**	1		z							
Sex	−0.106**	−0.047*	1								
HL total	0.366**	−0.045*	−0.023	1							
HL_finding info	0.384**	−0.052*	−0.020	0.936**	1						
HL_understanding Info	0.364**	−0.066*	−0.020	0.947**	0.859**	1					
HL_evaluating Info	0.343**	0.016	−0.040	0.927**	0.823**	0.837**	1				
HL_applying info	0.269**	−0.076**	−0.002	0.909**	0.790**	0.836**	0.782**	1			
Health behaviour	0.128**	−0.009	−0.235**	−0.142**	−0.120**	−0.135**	−0.098**	−0.188**	1		
Physical symptoms	−0.456**	0.148**	0.143	−0.370**	−0.406**	−0.374**	−0.313**	−0.281**	0.028	1	
Well‐being	−0.023	0.076**	0.059**	0.253**	0.218**	0.218**	0.225**	0.285**	−0.203**	−0.057**	

Note

Edu = education (0= ‘Primary school or lower’, 1= ‘secondary school or above’), marital status (0 = single/divorced/widowed; 1 = married), gender (0 = male,1 = female), HL = health literacy, HL_finding info = Health literacy in finding information, HL_understanding info = health literacy in understanding information, HL_evaluating info = health literacy in evaluating information, HL_applying info = health literacy in applying information; higher score of ‘health behaviours’ indicated more presence of unhealthy behaviours.

* *P* < .05;

** *P* < .01;

*** *P* < .001.

In consistent with previous literature, health literacy capacities have direct effects on health outcomes. After adjusting for education, age and marital status, higher level of HL in finding and understanding information was associated with fewer physical symptoms (HL_finding information: β= −0.569, *P* < .001; HL_understanding information: β= −0.385, *P* = .001); and higher level of HL in applying information was associated with greater subjective well‐being (β = 2.564, *P* < .001). In addition, health behaviour (indicated by the number of unhealthy behaviours) was also found to mediate the effect of health literacy on physical health and well‐being. In particular, the capacities of finding and applying health information was associated with fewer unhealthy behaviours (HL_finding information: β = −0.180, *P* = .07; HL_applying information: β = −0.198, *P* = .05), with the effect of HL_applying information being moderated by sex (β = −0.333, *P* = .01). More unhealthy behaviours were related with more physical symptoms (β = 0.118, *P *< .001) and lower subjective well‐being (β = −0.729, *P *< .001).

To probe the moderating effect of sex, the indirect effects of health literacy in applying information were compared between men and women. The results showed that among men, the capacity of applying information has marginally significant indirect effects on physical health and well‐being (physical health: β= ‐ 0.023, *P* = .088; well‐being: β = 0.144, *P* = .067). However, among women, the indirect effects on physical symptoms and well‐being were both significant, such that via reducing unhealthy behaviours, the capacity of applying information was associated with fewer physical symptoms (β= −0.063, *P* = .001) and greater well‐being (β = 0.387, *P* < .001). The details of the moderated mediation models were presented in Tables 3 and 4.

**Table 3:** The moderated mediation model of health literacy in predicting physical symptoms

	B (SE)	p‐value
Association between HL dimensions and Physical symptoms (DV: Physical symptoms ^a^; IV: HL dimensions)		
HL_finding information	−0.569***(0.102)	<0.001
HL_understanding information	−0.385**(0.116)	0.001
HL_evaluating information	0.113 (0.092)	0.220
HL_applying information	0.196 (0.102)	0.193
Association between HL dimensions and health behaviours (DV: health behaviours ^a^; IV: HL dimensions; moderator: sex)		
HL_finding information	−0.180 (0.103)	0.070
HL_understanding information	−0.083 (0.119)	0.478
HL_evaluating information	0.144 (0.096)	0.125
HL_applying information	−0.198* (0.106)	0.047
HL_finding information × sex	0.160 (0.138)	0.237
HL_understanding information × sex	0.003 (0.159)	0.984
HL_evaluating information × sex	0.059 (0.126)	0.631
HL_applying information × sex	−0.333** (0.131)	0.010
Association between health behaviours and physical symptoms (DV: physical symptoms ^a^; IV: health behaviours)		
Health behaviour	0.118*** (0.030)	<0.001
Indirect effects of HL dimensions via health behaviours in men and women (DV: physical symptoms ^a^; mediator: health behaviours)		
Men: HL_finding information	−0.021 (0.013)	0.108
HL_understanding information	−0.010 (0.014)	0.485
HL_evaluating information	0.017 (0.012)	0.155
HL_applying information	−0.023 (0.014)	0.088
Total effect	−0.583*** (0.056)	<0.001
Women: HL_finding information	−0.002 (0.017)	0.829
HL_understanding information	−0.010 (0.019)	0.415
HL_evaluating information	0.024* (0.015)	0.035
HL_applying information	−0.063** (0.020)	0.001
Total effect	−0.597*** (0.056)	<0.001

Abbreviations:

B, regression coefficient; DV, dependent variable of the underlying regression model; HL, Health literacy; IV, independent variable of the underlying regression model; SE, Standardized error.

^a^ Physical symptom was adjusted for age, education and marital status.

* *P* < .05;

** *P* < .01;

*** *P* < .001

**Table 4:** The moderated mediation model of health literacy in predicting well‐being

	B (SE)	*P*‐value
Association between HL dimensions and well‐being (DV: well‐being ^a^; IV: HL dimensions)		
HL_finding information	0.399 (0.370)	.280
HL_understanding information	−0.632 (0.420)	.133
HL_evaluating information	0.283 (0.332)	.395
HL_applying information	2.564*** (0.368)	<.001
Association between HL dimensions and health behaviours (DV: health behaviours ^a^; IV: HL dimensions; moderator: sex)		
HL_finding information	−0.180 (0.103)	.070
HL_understanding information	−0.083 (0.119)	.478
HL_evaluating information	0.144 (0.096)	.125
HL_applying information	−0.198* (0.106)	.047
HL_finding information × sex	0.160 (0.138)	.237
HL_understanding information × sex	0.003 (0.159)	.984
HL_evaluating information × sex	0.059 (0.126)	.631
HL_applying information × sex	−0.333** (0.131)	.010
Association between health behaviours and well‐being (DV: well‐being ^a^; IV: health behaviours)		
Health behaviour	−0.729*** (0.109)	<.001
Indirect effects of HL dimensions via health behaviours in men and women (DV: well‐being ^a^; mediator: health behaviours)		
Men: HL_finding information	0.131 (0.076)	.088
HL_understanding information	0.061 (0.087)	.480
HL_evaluating information	−0.105 (0.076)	.136
HL_applying information	0.144 (0.081)	.067
Total effect	2.845*** (0.204)	<.001
Women: HL_finding information	0.014 (0.103)	.828
HL_understanding information	0.063 (0.116)	.408
HL_evaluating information	−0.149* (0.093)	.018
HL_applying information	0.387*** (0.107)	<.001
Total effect	2.931*** (0.204)	<.001

Abbreviations:

B, regression coefficient; DV, dependent variable of the underlying regression model; HL, Health literacy; IV, independent variable of the underlying regression model; SE, standardized error.

^a^ Well‐being was adjusted for age, education and marital status.

* *P* < .05;

** *P* < .01;

*** *P* < .001.

## 4 DISCUSSION

Health literacy was found to be a key contributor to individual's health. Although the related changes in health behaviours were proposed to be an underlying mechanism of the health literacy, limited evidence has been found regarding the mediating role of health behaviour. By conducting a large‐scale survey across different age, the current study has tested a moderated mediation model of health literacy predicting physical health and subjective well‐being through influencing health behaviours. Furthermore, we have looked at the effects of specific health literacy skills, that is finding, understanding, evaluating and applying health‐related information.

In a sample of 2236 adults, we found the prevalence of limited HL was 55%, which was close to the average levels in Malaysia and Singapore.^5^ The prevalence of limited HL was higher among women than men, which was inconsistent with the previous findings that men actually have lower level of HL.^26^, ^39^ This sex difference might be driven by that men's education level was higher in our sample (χ^2^ = 24.99, *P* < .001), which contribute to higher health literacy. In fact, with logistic regression, it showed that although female are 1.19 times more likely to have limited HL than male (*P* = .04), when education entering the model, the sex difference became insignificant, and people with lower education are 2.47 times more likely to report limited HL (*P* < .001). It is also possible that men may use over‐report when answering health literacy questions, thus leading to a higher HL score.^28^ The structural modelling analysis showed similar patterns of health literacy in predicting physical health and subjective well‐being, while different health literacy capacities had different direct and indirect effects. HL in finding and understanding information showed a direct effect in predicting fewer physical symptoms, and applying information HL was directly associated with greater well‐being. As for the indirect effect via health behaviour, only health literacy in applying information showed a significant result, which was moderated by sex. Despite the patterns were similar, the indirect effect of the capacity in applying information on promoting health and well‐being was only significant among women.

This is the first study to test how the direct and indirect effects of health literacy vary across four dimensions. In line with a previous study on college students showing that understanding and applying health information had stronger effect in promoting people's smoking cessation,^40^ our findings suggested that finding and understanding information were associated with fewer physical symptoms, while applying information showed a mediation effect moderated by sex, that only among women the indirect positive effect reached significance. The results suggested that the capacities of finding and understanding information may influence our health in a more direct and general way, while the applying health information may function via modifying our behaviour, particularly for women. It is probably because applying information (eg, ‘make decisions to improve your health’, ‘Join a sport club or exercise class’) is usually the last step when making health decision, which is more closely related to taking action. Therefore, this is the only domain having an indirect effect on health via behaviours. Interestingly, the capacity of evaluating information didn't show any effect on physical symptoms or well‐being, which is consistent with previous findings that smoking cessation was associated with various HL capacities except evaluating information.^23^ It is possible that evaluating information was usually perceived as the most difficult (eg,,^6^, ^41^ in other words, it was a more advanced capacity and would not show immediate effects on individual's health behaviours or outcomes.

The mediating role of health behaviours is consistent with the previous pathway model linking health literacy to health outcome in the patient population, which suggested that health literacy functions mainly in three domains, including the access to and use of health care, patient–provider interaction and patient's self‐care.^42^, ^43^ The health behaviours fell into the domain of self‐care. Previous studies showed that health literacy is associated with being physically active, taking balanced diet and low usage of tobacco and alcohol.^22^, ^44^ By taking a further step, our results suggested that across different age groups, health literacy, especially better ability in applying health information, would reduce unhealthy behaviours and promote self‐care agency thus benefiting physical health and subjective well‐being. Moreover, this indirect effect of health literacy was only found among women, potentially indicating that with sufficient health information, women could more readily apply it to modify their behaviours and adopt a healthier lifestyle. Men, on the other hand, may need more motivations to transform the health information they have into actions.

Though our findings provided evidence for how health literacy influences health status and well‐being through behaviours, there are several limitations to be acknowledged. First, the health was measured by self‐reported physical symptoms, which could be influenced by individual's personal reporting styles, and may not accurately capture the health status of the individual. Future studies should adopt more objective measures, such as medical record or physiological measures to see if the influences of health literacy will still be found. Second, only smoking, drinking and physical exercise were included to evaluate people's health behaviour, and to have a more comprehensive measure of health behaviour, body examination or dietary habit, may also be included in future studies. At last, like most of the existing literature, only cross‐sectional data were collected in the current study, which may not be sufficient to support the causal relationship between health literacy and health outcomes. In other words, there might be a reciprocal association between the two. For example, a previous study has found that people with chronic disease may have higher level of health literacy, due to the needs for self‐care and disease management.^7^ Therefore, longitudinal data are necessary to further clarify the interplay between health literacy and health outcomes.

## 5 CONCLUSION

To conclude, the current study provided evidence suggesting that promotions for health literacy are still urgent for Hong Kong population, such that over 50% of the lay public showed a limited level of health literacy, and this prevalence was a little higher among women. In addition, by addressing specific direct and indirect effects of four dimensions of health literacy on physical health and subjective well‐being, the findings showed that better perceived capacities in finding and understanding health information could lead to better physical health, while greater capacity in applying health information is associated with healthier lifestyle. Based on the findings, more educational programmes to advocate health literacy and increase the awareness of healthy lifestyle should be developed for the lay public. In particular, skill trainings for finding and understanding health information should be provided to promote both men and women's physical health; while for women, it is important to enhance the ability to apply health information to behaviour modifications, which may further benefit their health and well‐being.

## 6 ETHICS APPROVAL AND CONSENT TO PARTICIPATE

The ethical approval for the study was obtained from the Human Research Ethics Committee of The University, and participants have provided written consent before taking part in the study.

## 7 CONSENT TO PUBLISH

Not applicable.

## 8 COMPETING INTERESTS

There was no competing interest that needs to be declared.

## CONFLICT OF INTEREST

The manuscript is not under simultaneous consideration elsewhere, and all authors have no conflict of interest to declare.

## AUTHORS' CONTRIBUTIONS

ZF has analysed and interpreted the data, as well as written and edited the manuscript. PO was a major contributor in generating the research idea, collecting data and editing the manuscript. JC was also a contributor in generating the research idea and data collection. All authors have read and approved the final version of the manuscript.

## Data Availability

Derived data supporting the findings of the study are available from the corresponding author on request.
